# A Concise History of the Principal Discoveries in Anatomy

**Published:** 1800-02

**Authors:** 


### *A conciſe Hiſtory of the principal Diſcoveries in Anatomy.*

[Continued f.om our laſt Number, pp. 72—79.

§ 29. THE knowledge of the *organs of generation*, and their functions, was conſiderably promoted in the ſixteenth century, by the united exertions of the moſt eminent anatomiſts; though they were ſtill unacquainted with many particulars. For inſtance, with reſpect to the external male organs of generation, the origin of the *corpora cavernoſa penis* was not quite familiar even to EUSTACHIUS, as he derived them from the urinary bladder and the proſtrate gland, inſtead of deducing them from the arcus oſſium pubis.

The greateſt anatomiſts of the age were much miſtaken with reſpect to the tunica albuginea teſtis. They were of opinion, that this tunica was actually connected with the abdomen by an orifice which was conſtantly open.† Though this holds good

---

* *Euſtach.* Tab. xi. fig. 11. (*KK.*)

† *Veſal.* lib. v. c. 13. p. 449.—*Fallop.* inſtit. anat. p. 439.

good in the embryo, it is not discoverable in a new-born infant. About the 20th day after birth, the sides of the orifice so firmly adhere, that no aperture is distinguishable. To other authors it appeared, that in the descent of the testicles only one of the lamina of the peritoneum passed down, to form the tunica albuginea, and the other remained in the abdomen.* Vesalius had remarked that the white coat of the testicle was subservient to the transmission of certain ducts, and consequently appears to have observed the vascula Graafiana with accuracy.† Massa ‡ described the prostrate gland, and subsequent to him Vesalius § and Columbus.‖ The discovery of the *vesiculæ seminales* must be attributed to Fallopius.¶ Prior to him, Berengar had indeed given some account of the winding ducts, and the cellular texture, in which the vasa deferentia are convolved,** as likewise Stephanus seems to have known them;†† but both express themselves obscurely on the subject. Vesalius first acquired from Fallopius the knowledge of the vesiculæ seminales,‡‡ and Eustachius at the same time, gave a representation of them.§§ Rondelet likewise found them distinct in the dolphin.‖‖ Finally, they were accurately described by Varoli ¶¶ and Albert.***

With respect to the female organs of generation, Fallopius first distinctly described the clitoris, and its resemblance to the penis.††† But Vesalius considered this description extravagant, and as being merely the result of observations made on some anomalous cases.‡‡‡ Columbus also deserves censure, for expatiating too much on the nature of the clitoris, in order to introduce indelicate expressions.§§§ Eustachius appears to have been the first anatomist who represented the sphincter vaginæ.‖‖‖ It is singular that there should, even in this century, arise any dispute about the existence of the membrane which partly precludes the vagina in virgins, and which the ancients had called *hymen*. We find, however, that the opinions and sentiments of authors, concerning this part, widely differed. Achillini maintained the existence of a virginal

---

* *Fernal.* part. corp. human. descript. lib. i. c. 7. p. 40.

† *Vesal.* lib. v. c. 13. p. 448.

‖ Lib. xi. c. 13. p. 436.

¶ Obs. p. 419.

** Isagog. p. 186. Comment. in *Mundin.* f. 302. b.

†† *Stephan.* de dissect. p. 193.

‡‡ Exam. obs. Fallop. p. 816.

§§ Tab. xii. fig. 3. (*VV.*)

‖‖ De piscibus, lib. xvi. c. 8. p. 461. (fol. Ludg. 1551.)

¶¶ Anatom. lib. iv. c. 1. p. 87.

*** Histor. part. p. 68.

††† Obs. p. 420.

‡‡‡ Exam. obs. Fallop. p. 819.

§§§ Lib. xi. c. 15. p. 447.

‖‖‖ Tab. xiv. fig. 1. (*XX.*)

virginal hymen, but certainly did not know it, when he contended that it was placed in the orifice of the uterus.* FALLOPIUS described it first, and best of all the anatomists of this century;† but VESALIUS, who partly admitted its existence, described its fibres to be muscular, and observed, that the cases which had occured to him, were extremely rare.‡ PARE likewise assures us, that he never met with an *hymen*.§ By COLUMBUS we are informed, that this membrane seldom exists, and renders coition almost impossible, from its remarkable thickness.|| VAROLI decidedly disbelieves in the existence of the hymen; but understands by it, the præternatural *concretion* of the labia interna pudendorum.¶ DU LAURENS is of the same opinion, and supposes this membrane to be an organic disease, but never admits its existence in a natural state.** PINEAU, considers even the carunculæ myrtiformes of the inner labia, instead of the hymen, as an infallible proof of virginity.†† In short, FALLOPIUS was the only anatomist, who knew and described this part with sufficient accuracy.

§. 30. With respect to the uterus, its ligaments were not completely understood in the sixteenth century. GABRIEL ZERBI described, however imperfectly, the round ligaments;‡‡ and besides, like LE VASSEUR,§§ admits adhesions of the uterus to the kidneys, which, however, do not exist. LE VASSEUR also describes, though not completely, the broad ligaments. VESALIUS calls the round ligaments muscles, but represents their progress very erroneously; yet he seems to have been acquainted with the alæ vespertilionum, and their membranous expansion.|||| He censures GALEN, for having described an animal uterus instead of a human;¶¶ and yet, as SYLVIUS justly remarks,*** VESALIUS's representation is, in many respects, not conformable to human nature, as he admits a threefold disposition of muscular fibres in the uterus,††† and has committed various other errors. FALLOPIUS denominated the round ligaments cremasteres, and proved, in opposition to VESALIUS, that they were not muscles; he correctedly described their progress through the aponeurosis of the oblique

* Annot in Mundin, p. 4.    † Obs. p. 420.

‡ Lib. v. c. 14, p. 457.—De radic. chyn. p. 663.—Exam. obs. Fallop. p. 819.

§ Liv. xxiv. ch. 49. p. 624.—xxviii. p. 773.

|| Lib. xi. c. 15. p. 446.    ¶ Anatom. lib. iv. c. 4. p. 97.

** Hist. anat. lib. vii. qu. 13. p. 562.

†† De virginit. not. lib. i. c. 5. p. 48. (12. LB. 1641.)

‡‡ *Zerb.* anatom. p. 43.    §§ *Vassei* in anatomen. tab. p. 10.

|||| Lib. v. c. 15, p. 461.    ¶¶ De radic. chyn. p. 663.

*** Vesan. Column. depuls. p. 113.    ††† Exam. obs. Fallop. p. 818.

oblique defcending abdominal mufcles, their ronnd and excavated ftructure at their infertion, and their termination in the adipofe membrane of the mons veneris; he demonftrated that they were fometimes the origin of ruptures in females; but ftill compared them too much with the cremafteres of the tefticles, according to the old prevailing idea, that the female fex have internally all the male organs of generation. *

We are indebted to EUSTACHIUS for the firft true reprefentation of the human uterus, though even he omits the broad ligaments and the alæ vefpertilionum† belonging to that organ. COLUMBUS, merely following FALLOPIUS, defcribed the round ligaments, under the denomination of *proceffus uteri*;‡ but PICCOLHUOMINI gives a correct account of thefe, as well as of the other connections of the uterus with the contiguous parts.§ The *tubæ fallopianæ*, which till that time had been always confounded with the cornea uteri in animals, were firft properly diftinguifhed by FALLOPIUS, and called by the abovementioned name; he reprefented their inner flakey texture, as well as their undulatory windings, their external larger aperture, and the fimbriæ furrounding the ovaria, which he called female tefticles. He farther confidered them as vafa deferentia of the female femen, for he pretended to have frequently obferved true femen in them, but never in the ovaria.‖ Neverthelefs, PICCOLHUOMINI denied the exiftence of the tubæ in the human uterus.¶ The hypothefis of the ancients, that women fecerned femen as well as men, and that it was preferved in the ovaria, or female tefticles, received ftill greater confirmation from the affertion of COLUMBUS,** that he had pofitively obferved femen in the female tefticles, and exhibited it to many of his pupils. FALLOPIUS, in defcribing the ftructure of the ovaria, admits of veficles filled with a limpid or yellow humour, which ftrongly indicates that he was acquainted with the ova graafiana and the corpora lutea.†† VESALIUS likewife defcribed the veficular ftructure of the ovaria.‡‡ COITERUS demonftrated their exiftence in ruminating and other animals; but he admitted three membranes of the ovarium, one originating from the peritoneum, and forming a thin and loofe exterior furface, and loofely furrounding it externally, the fecond uniting the veficles, and the third

---

* *Fallop.* obf. p. 421.

† Tab. xiii. xiv. fig. 1. 2. 3.

‡ Lib. xi. c. 15. p. 447.

§ Anatom. praelect. p. 186.

‖ *Fallop.* obf. p. 421.

¶ Anatom. praelect. p. 195.

** Lib. xii. p. 453.

†† L. c.

‡‡ Exam. obf. Fallop. p. 810.

third he seems to have confounded with the fimbriæ of the tubæ fallopianæ.*

§. 31. Misled by zootomy, many of the ancient physicians admitted, even in the human uterus, of *cotyledones*, such as are formed by the placenta of different animals; but VESALIUS shewed, that this term had various significations, because the distended orifices of the veins, even in the unimpregnated state of the animal uterus, were thus denominated; which, however, could not apply to the human uterus.‡ A farther explanation of this subject was given by FALLOPIUS.† PUTEUS, with superficial arguments, vindicated the presence of the cotyledones against VESALIUS, in the human uterus; § but ARANZI totally denied their existence, unless the orifices of the vessels were to be so denominated; ‖ and FABRICIUS, therefore, adopted them in that sense. ¶ The connection of the infantile fœtal part of the placenta with the maternal, likewise engaged the attention of the anatomists of the age. ARANZI disputed all communication of the vessels, as had commonly been adopted.** DU LAURENS admitted, that there was no true inosculation of vessels, but yet there existed a contiguity among them, sufficient for effecting resorption;†† and FABRICIUS asserted an anastomosis of the vessels on both sides,‡‡

The old opinion, that the conception of girls took place on the left side, and of boys on the right, still found several advocates in this century, of whom we shall only mention BERENGAR. §§ He proposed a theory, which afterwards was vindicated by almost all the anatomists, even by FABRICIUS; namely, that the liquor amnii originated merely from the perspiration of the fœtus. ‖‖ This assertion, however, is particularly objected to, in consequence of the remark, that a similar liquor had been found in unimpregnated eggs, and that it existed in a greater quantity, accordingly as the embryo approached to evolution.¶¶ FALLOPIUS seems to allude to the *tunica decidua Hunteri*, when he speaks of the cotyledones; though it cannot positively be asserted that he really knew it.***

MASSA

* Obs. p. 124.

‡ Obs. p. 422. f.

† Lib. v. c. 16. p. 467.—De radic. chyn. 645.

§ Apolog. f. 164. b.

‖ De fœtu human. c. 2. p. 5.

¶ De format. fœtu, p. 40

** L. c. c. 10. p. 28.

†† Lib. viii. qu. 24. p. 664.

‡‡ L. c. p. 38, 43.

§§ Commentar. in Mundin, f. 220. b.

‖‖ *Berengar*, l. c. f. 261. *a.*—*Fabric.* de format. fœtu, p. 37. 91.

¶¶ *Diemerbroek.* anatom. lib. I. c. 31. p. 212.

*** Obs. p. 423.

—— Massa,* Sylvius,† and even Vesalius,‡ defended the exiſtence of a third peculiar membrane, denominated *allantois* by the Greeks, and of which it was conjectured, that in the human embryo, as well as in the animal, it contained the urine of the fœtus, which it received from its urinary bladder by way of the urachus, as a true canal; nay, Puteus aſſerted, that he had ſeen a human allantois replete with excrements.§ Fallopius was, in this inſtance alſo, the firſt who preferred obſervation from human nature to all the inferences drawn from comparative anatomy. He proved, that the urachus in the human fœtus did not terminate in a particular membrane, but between the chorion and amnion.‖ Yet, he was miſtaken in aſſerting, that the urine in the human fœtus was evacuated through the urachus, and flowed into the intermediate ſpace between the amnion and chorion; that the latter membrane is provided with veſſels in animals only, and not in the human fœtus; and hence, the urine could accumulate only in the latter, as in animals it would corrode the veſſels of the chorion. Eustachius ¶ went ſtill farther in aſſerting, that there exiſted neither an allantois nor an aperture of the urachus in the human fœtus. Varoli endeavoured to confirm this aſſertion by new arguments;** but Fabricius, though he diſbelieved the exiſtence of the allantois in the human fœtus, he admitted of an opening of the urachus between the chorion and amnion, and maintained with Fallopius, that this place was deſtined as a reſervoir for the urine of the human fœtus.†† Fabricius has certainly not conſulted Nature, or he would have diſcovered, that the intermediate ſpace between the two laſt mentioned membranes, on account of the expanſion of the amnion, is in the ſecond month of pregnancy ſo ſmall, that it would admit ſcarcely any thing but a highly rarified vapour.

The anatomiſts of this century likewiſe made remarkable obſervations on the evolution of the embryo. Coiterus diſcovered in a ſow, on the tenth day after conception, a vitreous ſubſtance, encloſed in a membrane, which contained an embryo with diſtinct blood veſſels.‡‡ Varoli obſerved, on the twentieth day after conception, an embryo, of the ſize of a barley-corn, but of a form not diſſimilar to a kidney-bean.§§

[To be continued in our next Number.]

* Introduct. f. 15. a.

† Iſagog. p. 13.

‡ Lib. v. c. 17. p. 470.

§ Apolog. f. 155. b.

‖ Obs. p. 424.

¶ Off. exam. p. 204.

** Lib. iv. c. 5. p. 113.

†† De format. fœtu, p. 91. f.

‡‡ *Coiter*. obſ. p. 124.

§§ Anatom. lib. iv. c. 4. p. 102.

